# Micellar-Enhanced Ultrafiltration Using a Plant-Derived Surfactant for Dye Separation in Wastewater Treatment

**DOI:** 10.3390/membranes10090220

**Published:** 2020-09-02

**Authors:** Nita Aryanti, Aininu Nafiunisa, Tutuk Djoko Kusworo, Dyah Hesti Wardhani

**Affiliations:** 1 Department of Chemical Engineering, Diponegoro University, Semarang 50275, Indonesia; aininunafiunisa@gmail.com (A.N.); tdkusworo@che.undip.ac.id (T.D.K.); dhwardhani@gmail.com (D.H.W.); 2 Membrane Research Centre (MeR-C), Diponegoro University, Semarang 50275, Indonesia

**Keywords:** dye solubilization, blocking mechanism, membrane filtration, saponin

## Abstract

Micellar-enhanced ultrafiltration (MEUF) is one of several membrane methods used for the removal of trace organic pollutants from aqueous streams. In this process, a surfactant is added to a polluted aqueous solution at a concentration higher than its critical micelle concentration (CMC). Unlike synthetic surfactants, natural surfactants, from plants such as the saponin, while ecologically adaptable as surfactants in MEUF systems, are also biodegradable, renewable, and environmentally safe. This study applied Sapindus rarak extract as the natural surfactant in MEUF for Remazol dye separation. It was found that the presence of Sapindus rarak extract increased separation of Remazol red and blue dyes by up to 97.02% and 99.42%, respectively. However, the addition of surfactant decreased permeate fluxes due to membrane fouling and concentration polarization. In addition, loading micelle (Lm), representing the performance of the surfactant micelle for dye separation, as well as the blocking mechanism, was investigated. Lm was found to be in the range of 0.002–0.068 mM dyes/mM saponin. Ultrafiltration blocking mechanisms, as confirmed by the Hermia model, were: standard blocking, for cases without the addition of surfactant; cake formation, for cases with surfactant below the CMC; and complete blocking, for cases with surfactant above the CMC.

## 1. Introduction

**Figure 1:**
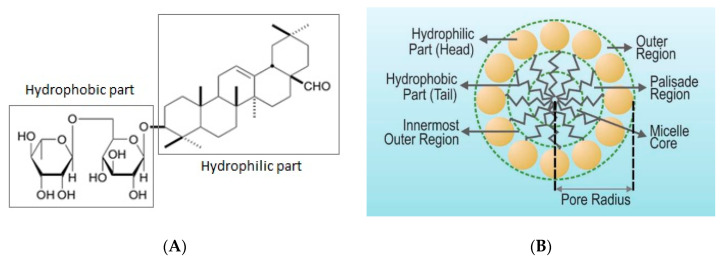
(**A**) Part of saponin; (**B**) Part of surfactant micelle in water.

Reactive dye pollutants are widely generated nowadays due to the rapid growth of various dyes involved in industries such as paper printing, plastics, cosmetics, and the textile industry. Most of the time, the reactive dye pollutants are discarded into the water body without appropriate treatment, resulting in significant environmental issues [1]. The treatment of dye effluents by membrane technology, especially ultrafiltration, is not easy due to the small size of the dye pollutants and the high water solubility of the dyes. Modification of the ultrafiltration system, namely Micellar-enhanced ultrafiltration (MEUF), is a superior method of removing traces of dangerous pollutants, such as dyes, from water-based effluent [2]. MEUF utilizes filtration by membrane and micellization of a surfactant. It has a high rate of rejection in nanofiltration and reverse osmosis as well as a high flux of ultrafiltration. The basic principle of MEUF is that a surfactant forms large amphiphilic micelles when added to an aqueous stream at a concentration higher than its critical micelle concentration (CMC). The CMC is the concentration where micelles of the surfactant start to form [3]. This micellar formation causes a solubilization effect before the ultrafiltration process. A micellar solubilization occurs when the solute, in relation to its affinity in the aqueous medium, is located inside the micelle. The H-bonding or dipole–dipole attraction between the polar groups of the dyes and surfactant is the presumable mechanism [4]. The size of micelles that contain solubilized solute are large, with an aggregation number in the range of 50 to 100 [2]. As a result, the solutes entrapped in surfactant micelle are rejected by the ultrafiltration membrane. In general, micelles of surfactant are involved in the inner core, palisade layer (constituted by CH_2_ groups) and outer layer [5]. The inner core of the saponin is constituted by hydrophobic aglycone groups, while the outer layer is constituted by hydrophilic sugar groups. The illustration of the surfactant micelles part of saponin is presented in Figure 1.

Surfactant-enhanced remediation technology has shown potential in removing various organic and/or inorganic residual compounds from the liquid effluent. MEUF has been studied for its potential to remove various pollutants such as heavy metal ions, phenolic compounds, hydrophobic organic chemicals, and also reactive dyes. As for reactive dye removal, the method has been successfully applied in the removal of indigozol dyes [6], methylene blue [7], eriochrome blue black [4], a reactive black and orange mixture [8], and eosin [9].

Although MEUF is known to be an effective method in the removal of dye pollutants from wastewater effluent, the potential problem of toxicity of the synthetic surfactant remains. Most commonly-used surfactants are chemically synthesized, such as hexavalent trimethyl ammonium bromide (CTAB), sodium dodecyl sulfate (SDS), hexadecylpyridinium chloride (CPC) [10,11], Tween 80, Brij, Marlophen NP5 [2], and various other surfactants. The degradation process of synthetic surfactants is slow. Some are persistent in the environment and may cause estrogenic activity and chronic toxicity. In MEUF, the surfactant remains in a retentate stream at a high concentration, which creates secondary pollution [12]. Therefore, before disposing of them into the water body, a post treatment process is needed, which incurs additional costs in the process. The use of synthetic surfactant is also considered to take up a large portion of costs, so some studies have proposed additional methods to recover the synthetic surfactant from the retentate solution [13]. Thus, the selection of the surfactant is very important and must not be overlooked. Natural surfactants, such as saponin, are a good choice as an alternative to synthetic surfactants. Saponins are easy to find and can be extracted from various plant sources such as *Sapindus rarak DC*, which is widely found in Southeast Asian countries [14]. By using saponin in the MEUF process, the post treatment such as surfactant recovery from the retentate is not required, because natural plant-based surfactants such as saponin are naturally degradable [15]. 

This study emphasized the application of a natural surfactant in the micellar-enhanced ultrafiltration membrane for removal of reactive dye pollutant. The design of the study focused on the removal of two types of Remazol dye (Remazol Red RB, and Turquoise Blue), which are widely used in coloring textiles. The two dyes represent the common characteristics of reactive textile dyes having small molecular weight and forming a colloid in aqueous solution. Previous reports of MEUF incorporating a natural surfactant have been scarce: the only such study found by the author used reetha soapnut for the removal of only one kind of dye, methyl violet [12]. The study investigated the solubilization power and membrane performance. However, the micelle loading and blocking mechanism is not investigated yet. Moreover, a comprehensive study of the direct application of saponin extract from *Sapindus rarak DC* is very limited. In addition, only a few investigations were found to study the other dye type. Moreover, membrane performance and the dye solubilization were also investigated in the current study. The membrane performance investigation included the flux profile of various dye wastewater effluents, and the rejection of dye pollutants and the saponin surfactant itself. The addition of surfactant in the MEUF process typically lowers the permeate flux due to the blocking of foulant in the membrane surface [3,7]. Membrane fouling generally occurs in forms of foulant adhesion/deposition [16,17] and thermodynamic filtration resistance of the foulant layer [18,19]. Hence, to understand the blocking mechanism, a blocking model was also tested, followed by a morphological analysis.

## 2. Materials and Methods 

**Figure 2:**
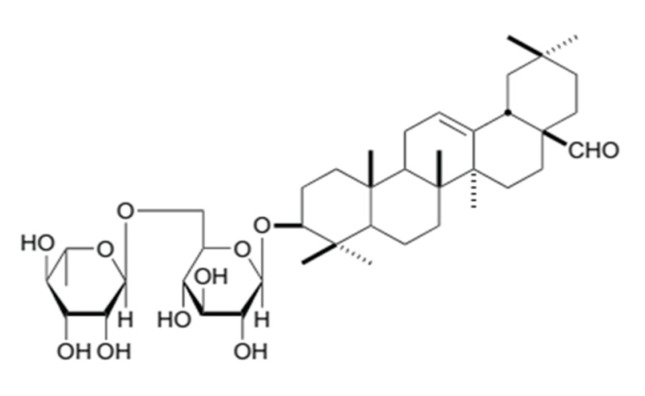
Molecular structure of saponin from the *Sapindus rarak DC*. extract [20].

The membrane used in this study was a flat sheet polyethersulfone membrane with a molecular weight cut-off of 10 kDa (Sterlitech, Kent, Washington). The feed solution was a dye wastewater model consisting of distilled water as the solvent and Remazol dyes (Red RB, and Turquoise Blue) (Nat Collection, Surakarta, Indonesia). Remazol dyes were added into an appropriate amount of distilled water to a concentration of 300 ppm. Extract of *Sapindus rarak* was used to replace the surfactant. The extract was prepared by ultrasonic-assisted extraction at 30 °C, with a ratio of solid to liquid of 1:10 (*w*/*v*), for 40 min. The raw pericarps of *Sapindus rarak DC* was purchased from Batik Zie, Semarang, Indonesia. The general molecular structure of the saponin extract is shown in Figure 2.

The saponin extract was added to the reactive dye wastewater in the suitable range of 0, 0.5, 1, 1.5 and 2 times the CMC content, with the CMC determined to be 3.075 mM. The determination of CMC was conducted by surface tension drop methods. The solution was then agitated using a magnetic stirrer at 200 rpm for 30 min to provide efficient mixing, and kept at a room temperature of 26 ± 1 °C to reach equilibrium [21]. Prepared feed solutions were then used for the MEUF experiments. Filtration was carried out using a filtration cell with cross-flow mode, equipped with a water pump and pressure control. In each run, the membrane was first placed in distilled water for 2 h before use. Further, it was compacted in the cell and compressed by up to 3 bars for approximately 1 h using distilled water [22]. Pure water flux (*J_w_*) was also measured for each run. To obtain a clear flux profile, permeate fluxes (*J*) were determined by weighing permeate collected every 5 min for 120 min [7]. Total operation time was determined by assuming that the flux steady state condition was achieved after 120 min. Permeate flux was calculated based on Equation (1):

(1) $$J\;=\;\frac{V}{A\cdot t}$$

where *V* is the permeate volume, *A* is the membrane area, and *t* is the interval of time. Ultrafiltration was performed without surfactant addition in the feed solution, and with the surfactant addition (model of surfactant solution), called the MEUF system. The experiment was conducted in a total recycle system. In this system, both permeate and retentate were recycled into the feed tank. Further, the permeate and retentate were collected and analyzed at 0, 30, 60, 90, and 120 min. Samples were then mixed into an average single solution. The data was then presented as the normalized flux (*J*/*J_w_*). Performances of UF and MEUF in removing dyes from the wastewater model solution were evaluated by dye rejection. The rejection was determined based on Equation (2):

(2) $$\%R=\left(1-\frac{C_{p}}{C_{f}}\right)\times 100\%$$

where *C_P_* is permeate concentration and *C_F_* is feed concentration. The concentration of the dye was determined using spectrophotometric methods at the maximum wavelength using calibration methods. A solution of each substance at pure condition was used in the calculation of the maximum wavelength. The analysis of each sample was measured simultaneously in mix component mode. The maximum absorbance (*λ_max_*) value for aqueous saponin was 277 nm. For the remazol dyes, Red RB and Turquoise Blue, the maximum absorbances were 521 nm and 663 nm, respectively.

To ensure the effect of surfactant micelle formation in the ultrafiltration process of dye removal, analysis of equilibrium distribution constant (*K_d_*) and micelle loading (*L_m_*) was conducted [7]. According to the law of mass action, one definition of *K_d_* is:

(3) $$K_{d}=\frac{D_{m}}{S_{m\;}\;-\;D_{w}}$$

while *L_m_* is defined as:

(4) $$L_{m}=\frac{D_{m}\;-\;D_{w}}{S_{m\;}\;-\;S_{w}}$$

where *S_m_* and *D_m_* are the total concentrations of surfactant and dye pollutant in the retentate, respectively, and *S_w_* and *D_w_* are the total concentrations of surfactant and dye pollutant in the permeate, respectively. Concentrations of dye in both retentate and permeate were analyzed using spectrophotometric UV-Vis with calibration methods. Saponin content was analyzed using the same tools and methods, only in the UV wavelength.

Analysis of the membrane fouling mechanism was conducted by applying a mathematical model to Hermia’s model. Hermia’s model provided a comprehensive fouling prediction model, well-equipped with four different fouling mechanisms [23]. The mechanisms of four blocking filtration laws, adapted for cross-flow filtration where trans-membrane pressure acted as the driving forces, were schematically illustrated in the previous study [24].

The model is expressed in Equation (5):

(5) $$\frac{d^{2}t}{dV^{2}}\;=\;k{\left(\frac{dt}{dV}\right)}^{n}$$

where *V* is the volume of filtrate collected in time *t*, and *k* and *n* are constants, dependent upon the mechanism involved. As analyses of membrane filtration are normally performed in terms of flux, it should be noted that Equation (5) can be presented in an alternative form, d*V*/d*t* = *A·J*, where *A* is the total membrane active area and *J* is the permeate flux, so that it can be rewritten in a physically more meaningful form as shown here in Equation (6):

(6) $$-\frac{1}{A^{2}\cdot J^{3}}\cdot \frac{dJ}{dt}\;=\;k\;{\left(\frac{1}{A\cdot J}\right)}^{n}$$

For complete blocking, it is assumed that each solute molecule arriving at the membrane surface participates in blocking by means of pore sealing, because of its size being bigger than the membrane pore. The big molecule tends not to settle over another molecule that has been deposited on the membrane surface. Thus, the fractional reduction in permeate flux is equal to the fractional reduction in the membrane surface area corresponding to unblocked pores, but not inside the membrane pores. The mechanism of complete block of some part of the membrane surface implies that the n value of the Equation (6) must take a value equal to 2 [24]. For standard blocking, the model considers that molecules pass into the membrane pores and deposit over the pore walls due to the variability of the pore opening, and reduce the volume of the membrane pore. As a result, the membrane pore volume decreases in proportion with the filtered permeate volume [25]. This decrease in the volume of membrane pores with time is equal to the decrease in their cross section. In this case, flux decline was expressed by letting n = 1.5. In comparison to the complete pore blocking model, the intermediate fouling or blocking model considered the probability that some molecules may settle over others [24]. Non-blocked membrane surface diminished with time, thus the probability of a molecule blocking a membrane pore declined continuously with time, until n = 1. As for cake fouling, the model considered that solute molecules did not enter the membrane pores, but formed a cake layer over the membrane surface. Flux decline was then represented by replacing n in Equation (6) with 0. After taking account of the value of n and the condition resulting from each fouling mechanism, the linearized equations were derived according to Equation (6), and the values of constant k for each equation were obtained from the slope of the line and are given in Table 1, with *K_c_*, *K_s_*, *K_i_*, and *K_ef_* as the constant of each type of blocking mechanism model, and *J*_0_ is assumed as the flux at the initial time. 

The modeling calculation of the blocking mechanism was performed using Ms. Excel. The determination of the fitted blocking mechanism model was represented by the value of the corresponding correlation *R*^2^. Moreover, to ensure the effectiveness of the mechanism of fouling on the membrane surface, a further analysis was conducted using scanning electron microscopy (SEM) and a FTIR analysis.

## 3. Results and Discussions

### 3.1. Profile of Permeate Flux in the Surfactant-Enhanced Ultrafiltration System 

**Figure 3:**
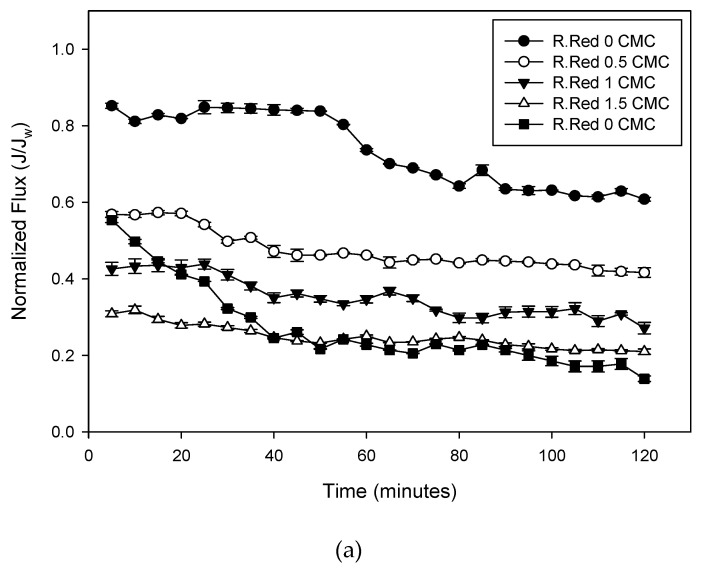

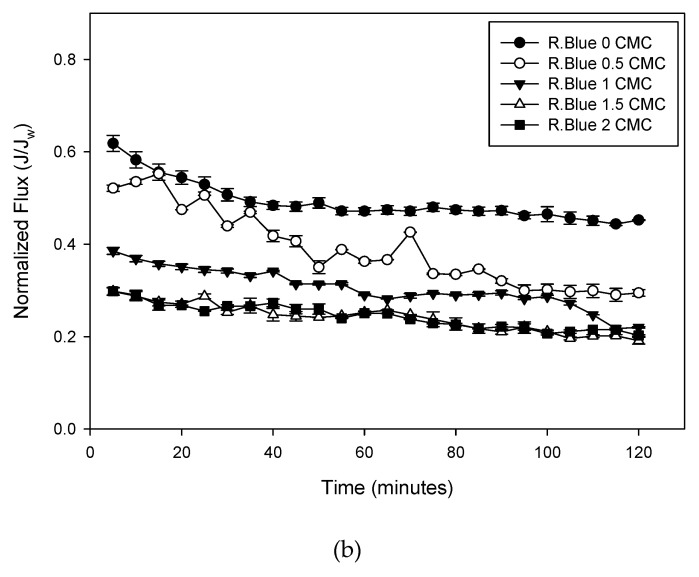
Variation of the observed permeate fluxes of (**a**) Remazol Red RB and (**b**) Turquoise Blue, at various saponin concentrations, with time, at room temperature, a pressure of 1.5 bars and a dye concentration of 300 ppm.

This study was conducted to observe the effect of adding a natural surfactant, saponin, to Remazol dye variants, on MEUF system performance, based on the flux profile. The wastewater was a model made of two types of reactive dye, Remazol Red RB and Remazol Turquoise Blue. In this study, the effect of saponin concentration on the flux profile was investigated. Flux profiles were compared for filtration and ultrafiltration processes with feed solutions, at various saponin concentrations. Flux profiles of each run are presented in Figure 3.

Figure 3 shows the observed normalized fluxes of Remazol Red RB and Turquoise Blue over time. Permeate flux for all variables slightly declined with processing time, and remained almost unchanging for the rest of the experiment. This flux (*J*) constant value was considered as the steady-state permeate flux. The phenomenon of flux decline was due mainly to the effect of concentration polarization and the membrane fouling [7]. Concentration polarization appeared as a result of a deposition of dye molecules close to the membrane surface. The deposition and accumulation of solutes on the membrane surface and also within the membrane pores resulted in a cake layer formation and pore blocking, which lead to a membrane fouling. With the addition of saponin, the surfactant micelles generated a deposited layer on the top of the membrane surface (concentration polarization). This increased the resistance against the solvent flux through the membrane. Consequently, in the early stage of the MEUF experiments, the micelles accumulated near the membrane surface, producing a sharp decline in permeate flux due to a complete blocking of the membrane pore by the surfactant micelle. As the filtration proceeded, the fouling layer became denser, leading to a slow flux decline. In the final stage, membrane fouling was fixed and the permeate flux reached its steady state value. A similar result was also found in a previous study using ionic and non-ionic surfactant to remove emerging contaminants [3] and cadmium ions [11] and to recover phenolic compounds [26]. Removal of various indigosol reactive dyes using the cationic surfactant SDS also showed flux profiles similar to the one in this study [6]; however, the previous study also stated that osmotic pressure and precipitation could also have contributed to the flux decline [4]. In the other study, the gradient of the osmotic pressure difference across the membrane (related to micelles concentration in permeate and retentate streams) reduced the effective trans membrane pressure and, as a result, permeate flux decreased with operating time [27].

As shown in Figure 3, with the absence of saponin, Remazol Red solution showed a more rapid flux decline at the end (Figure 3a), while Remazol Blue showed a rapid decline at the beginning of the UF process (Figure 3b). This phenomenon was associated with the molecular weight difference between the two dyes. The dyes’ molecular weights were 668.999 g/mol and 1098.062 g/mol for Remazol Red RB and Remazol Turquoise Blue, respectively. The Remazol Blue molecule is larger in size. It can attach and deposit on the internal wall of the membrane pores more than the Remazol Red RB. This pore blocking can produce a rapid decline in flux at the beginning of the process. However, flux then only slightly declined after 40 min of UF. There was the possibility that excess dye molecules were carried out again by the feed current. Meanwhile, the Remazol Red RB molecules were small, far smaller than the membrane pore, which was 10 kDa. In the UF process, the red dye molecule easily permeated the membrane pore, which showed a rather high and stable flux profile. However, deposition of dye molecules inside the membrane pore or surface was undeniable. After 60 min of filtration, dye molecules started to affect the flux profile: it declined rapidly at the end of the filtration process due to continuously blocked membrane pores. 

Figure 3 also shows the effect of added saponin on flux profiles. Flux of the feed solution, without any added saponin, was slightly higher than that of the feed with saponin. In general, there was a slight decrease in flux values with increasing saponin concentrations in the feed solution. This was because of the interaction between the saponin molecule and pollutant, where the pollutant was entrapped in the micelle saponin structure. An ionic reactive dye, such as Remazol, can be solubilized into hydrophobic and hydrophilic media in micelles, or dissociate to ions that are adsorbed on the surfactant micelles [28]. The addition of saponin (surfactant) to the feed solution at concentrations above the CMC generated the formation of surfactant micelles [23]. The pollutant matters get embedded in the micelles via ‘‘like dissolves like’’ principle, while heavy metal ions are adsorbed on the opposite-charged micelles via electrostatic interaction [29]. The dye pollutant molecule was trapped in the innermost part of the micelle hydrophilic outer region. These hydrophilic sides of the surfactant micelles had a tendency to attach to each other in solutions with lower water content [12]. The micelles formed a layer and blocked water from getting through the membrane, resulting in the lower flux. The layer then became constant after reaching equilibrium. On both types of dye, the addition of saponin to a concentration higher than its CMC showed similar results. Solutions with saponin added at 1.5–2 × CMC showed a similar normalized flux. This indicated that the addition of excess saponin would not generate more blocking in the membrane.

### 3.2. Rejection of Dye and Saponin Residue

Membrane ultrafiltration performance is determined by its ability to retain a particular component. This is defined as the value of the rejected compound divided by the initial compound concentration, and identified as % rejection [22]. Table 2 shows the impurity concentrations on the permeate after filtration.

Based on Table 2, for both Remazol Red and Blue, dye concentrations in the permeate of the UF system were higher than in the MEUF. This was reflective of the phenomenon where more dye impurities transfer into permeate in the ultrafiltration system, whether caused by direct pass-through of the membrane film or convective transfer of solute particles. Addition of surfactant to the polluted wastewater resulted in reduced impurity concentrations on the permeate. As the saponin was added at 0.5 × CMC, dye concentrations slightly decreased from 187.73 to 148.58 ppm and 170.75 to 100.03 ppm for Remazol Red and Blue dyes, respectively. This showed that the addition of saponin below the CMC also affected dye rejection. It was not anticipated that the permeate dye concentrations would decrease, since it has often been thought that surfactant monomers did not form micelles while surfactant concentration was below the CMC value. However, due to the concentration polarization effect, the fouling layer formed at the membrane–bulk solution interface. In the fouling layer, the concentration of saponin may have exceeded the CMC value while the concentration of saponin on the rest of the bulk solution decreased, thus the surfactant micelles would have formed [5]. The formation of micelle could have resulted in weak solubilization and the ability to reject some of the dye pollutant. At the same time, high concentrations of saponin in permeate were also found. This showed that most of the saponin was present as free monomer molecules. Considering its size, the monomer molecule would be expected to pass through the membrane pore easily.

The addition of saponin equal to the CMC value drastically decreased dye concentrations, from 148.58 to 10.34 ppm and 100.03 to 3.83 ppm for Remazol Red and Blue, respectively. At CMC, the saponin started to aggregate and form micelles, resulting in the reduction in dye concentrations. Moreover, the saponin concentration on permeate also decreased, showing that the saponin started to grow in size and could be retained by the membrane. Dye concentrations in the permeate continued to decrease with increasing saponin. Obviously, with the incremental increase in saponin concentration, more micelles would aggregate and more dye molecules would be solubilized in the micelles. As explained in the previous section (Section 3.1), the dye could attach to the surfactant micelle by the mechanism of hydrophobic and hydrophilic properties of the dyes and the surfactant micelles, allowing the molecule to grow in size. The previous study presented that the aggregation number of saponin was around 13–21 [30], with a molecular weight of 1223.3 g/mol. It means that a micelle of saponin can grow its size to at least 10 times its original molecular weight. Compared to the MWCO of the membrane, which 10 kDa, it can be considered that the size of the micelle was bigger than the membrane pore size. This demonstrated that the dye pollutant, which embedded on the surfactant micelle, can be retained by a high MWCO (molecular weight cut-off) ultrafiltration membrane. A previous study also investigated MEUF to treat wastewater using ionic and non-ionic surfactants [9]. Successful results have been reported on the removal of cadmium ions using SDS and mixed surfactant [11], boron ions [31], and zinc ions [32].

**Figure 4:**
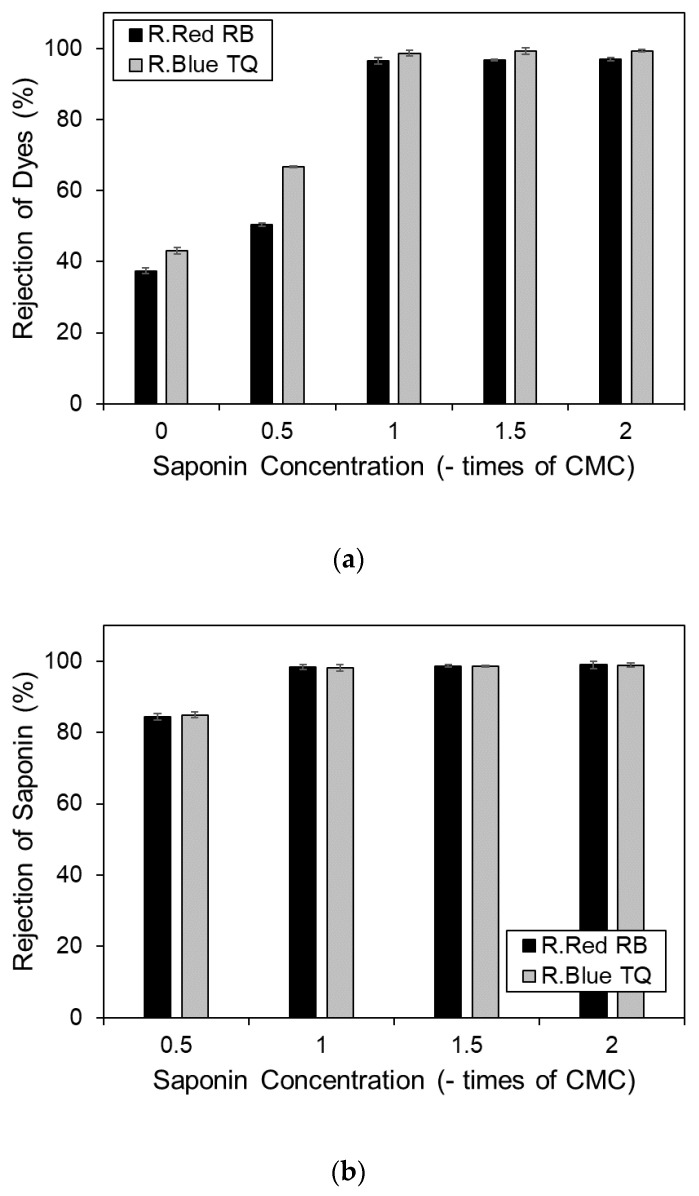
Rejection of Remazol dyes at various saponin concentrations (0, 0.5, 1, 1.5, and 2 × CMC), (**a**) rejection of dye, (**b**) rejection of saponin.

Pollutant concentration on permeate also affects membrane rejection. Permeate with a lower impurity concentration produces better membrane rejection. Membrane rejections of various Remazol dye filtrations under ultrafiltration and MEUF are shown in Figure 4. These results conformed with the known trend of impurity concentration on permeate: increased surfactant concentration will increase dye and saponin rejection. Here, MEUF showed a superior result compared to the UF system with higher rates of rejection of the dyes, 97.02% and 99.42% for Remazol Red and Blue, respectively, at a saponin concentration of 2 × CMC. The UF system was only able to achieve rejection rates of 37.42% and 42.08%.

### 3.3. Performance of Surfactant in the MEUF System: Micelle Loading an Equilibrium Distribution Constant

In this study, most of the solute in the dye-containing effluent (dye molecule) was solubilized in the micelles. Micelles containing solubilized solute were larger in size, making for easier filtration by the ultrafiltration membrane. For this reason, the ability of surfactant to solubilize the pollutant molecule was very important. In this study, surfactant performance was predicted by calculating the micelle loading (*L_m_*) and the equilibrium distribution constant (*K_d_*). The effect of saponin concentrations on the *L_m_* and *K_d_* of the Remazol dyes is presented in Table 3. Variations in saponin concentration were calculated based on the CMC of Sapindus rarak saponin, which has been determined to be 3.075 mM.

The system feed was a pseudophase solution that contained dye molecules, free surfactant molecules, surfactant micelles, and surfactant micelles with entrapped dye molecules. In this solution, micelles were a dynamic aggregate at equilibrium with the individual surfactant molecules. Individual surfactant molecules could pass through the pores of a membrane along with the free dye molecule. Bielska et al. [28] stated that the distribution of the dye in the pseudophase (micellar and aqueous), in permeate and retentate, was in equilibrium. As a result, ultrafiltration could be used to estimate the distribution coefficients of dye molecules in the retentate and permeate. This coefficient is defined as the ratio of dye concentrations in the retentate and permeate.

In this study, a very low *L_m_* was found for the solution with added saponin below the CMC value, for both Remazol Red and Blue. This corresponded to the fact that under the CMC, there is not much micelle formation. Hence, only a very minute amount of dye solubilized in the saponin micelles. At the CMC, micelle loading increased significantly, to as much as 0.068 mM of Remazol Red RB loaded into 1 mM of saponin, and 0.055 mM of Remazol Turquoise Blue loaded into 1 mM of saponin. Further addition of saponin to 1.5 × and 2 × CMC led to a decrease in *L_m_* to 0.034 mM/mM and 0.043 mM/mM for Remazol Red and Blue, respectively. This phenomenon was not anticipated considering that as more surfactant is added into the dye solution, more dye will load into the micelles, thus increasing the *L_m_* [5]. However, in this study, the saponin was added to the MEUF system when other parameters should have been taken into account, such as the maximum solute capacity of the membrane. The membrane used in this study was a commercial standard with a suggested feed solute capacity of 180–300 ppm. Thus, the dye concentration in the feed for this study remained constant at 300 ppm for all variables. There was a possibility that this dye concentration would not need too much saponin to solubilize it. As known, micelle loading is the proportion of dye solubilized in the micelles to the concentration of surfactant that formed the micelles [33]. At saponin concentrations of 1.5 times and 2 times of CMC, the concentration of dye solubilized in the micelle was similar to the one at the saponin concentration right at CMC; however, the concentration of surfactant that formed the micelles was higher due to the addition of more surfactant. Hence, the *L_m_* was decreased after the further addition of saponin. This was also confirmed by the rejection value discussed in the previous section, which showed only an insignificant increase despite the addition of saponin to above its CMC.

According to Table 3, the equilibrium distribution constant continuously increased with the addition of surfactant regardless of the concentration remaining below the CMC. *K_d_* was defined as the ratio of dye concentration in the micelle to that in the surrounding water for a particular surfactant concentration [5]. Thus, the increase in *K_d_* was obvious, considering that, with the addition of saponin, the concentration of free dye in the solution decreased, while more dye molecules solubilized in the saponin micelles.

### 3.4. Model of The Fouling Mechanism

**Figure 5:**
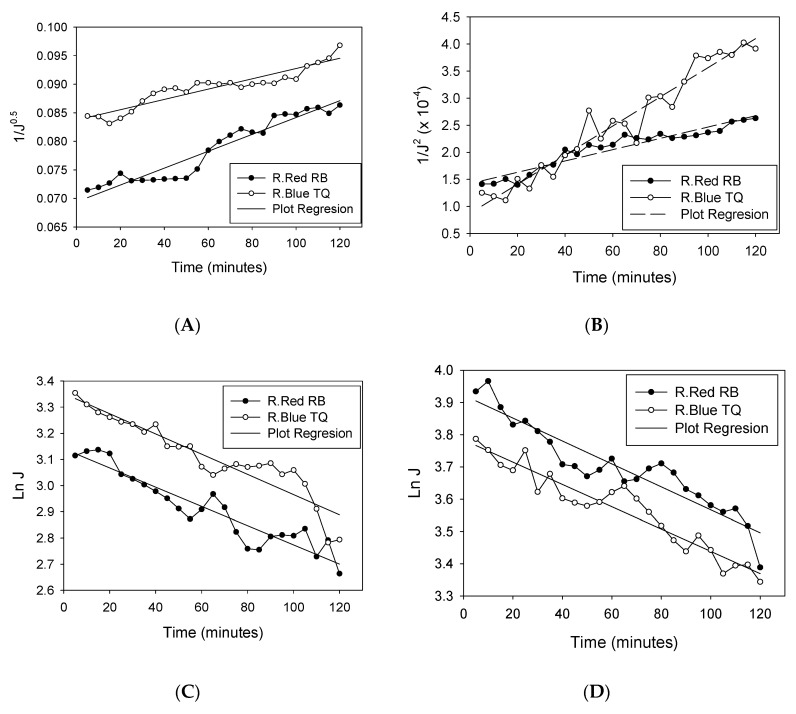

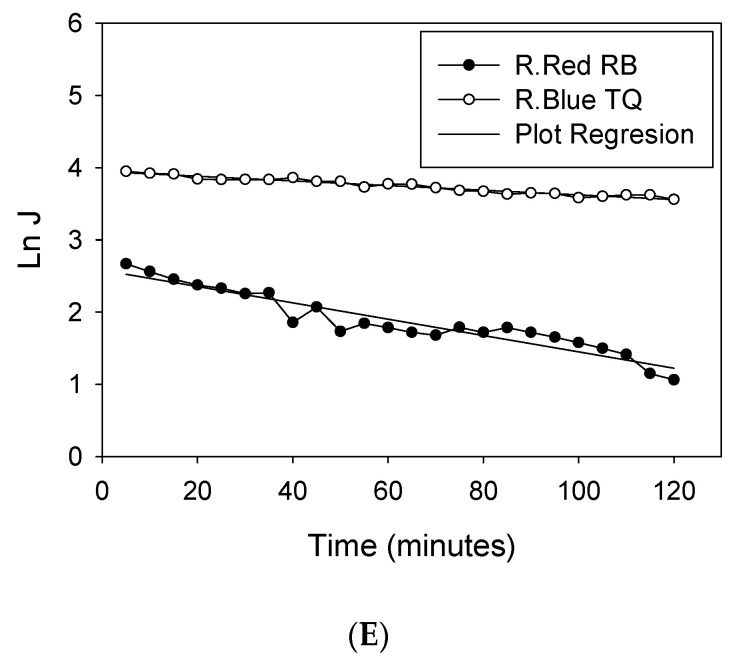
Fitting of experimental data to Hermia’s model for Remazol Red and Blue dyes at saponin concentrations of (**A**) No saponin (UF), (**B**) 0.5 × CMC, (**C**) 1 × CMC, (**D**) 1.5 × CMC, (**E**) 2 × CMC.

Mathematical models can be useful in accurately predicting the fouling phenomenon on the membrane filtration process. The blocking mechanism of Remazol dyes during ultrafiltration and MEUF was studied by applying Hermia’s mathematical model. Fitting experimental data based on Hermia’s model for each saponin concentration are presented in Figure 5, and the model parameter, as well as the degree of model fitness (represent by *R*^2^), is presented in Table 4. The value of corresponding correlation (*R*^2^) was simply used to determine the fitted blocking mechanism rationally. Bold values in the table show the best fitted fouling mechanism model with the experimental data. Based on the fitted model, the MEUF of Remazol Red RB and Turquoise Blue showed similar results.

The UF process without the addition of saponin fitted the fouling mechanism of standard blocking, with an *R*^2^ of 0.952 and 0.897 for Remazol Red and Blue, respectively. The molecular sizes of Remazol Red and Blue were far smaller than the membrane MWCO. This fact supported the phenomenon of dye molecules easily passing through the membrane pore. Some of the dye molecules got entrapped inside the membrane pore instead of escaping it. This resulted in the blocking of the membrane pore, which showed a standard blocking mechanism in this study. A previous study of MEUF for the synthesis of galacto-oligosaccharides also proposed that a solid molecule of molecular weight less than the membrane MWCO should cause standard blocking. This blocking process also follows from weak membrane rejection [34], which corresponded with this study. The fitted mechanism of the standard blocking for the feed without the addition of saponin also agrees with the discussion in point 3.1, which shows that the flux declined after a certain time instead of a rapid decline in the beginning of the filtration.

As presented in Table 4, the micellar-enhanced ultrafiltration of all Remazol dyes with saponin concentrations above CMC showed a fitting to a complete blocking mechanism, with a range of *R^2^* from 0.876 to 0.975. Complete blocking, by definition, results in a reduction in open pores without deposition of foulant particles on the membrane surface. This blocking occurs when the foulant particle size is similar to or larger than the membrane pore size [35]. As explained above, the addition of saponin above the CMC will generate molecular growth because of micelle formation. Dye molecules will grow in size until they are larger than the membrane MWCO, resulting in a complete blocking of membrane pores. However, this molecule was not expected to develop a cake layer, yet only expected to form, at most, a “flowing cake” layer or a very small (or nil) “stagnant cake” layer on the membrane surface. The large molecules of solubilized dye only blocked the pore membrane due to the pressure caused by the feed flow and did not generate a cake layer. This finding corresponds to the discussion in point 3.1 where the decline of flux profile occurred in the beginning of filtration and continued with a rather stagnant flux along the filtration. The solubilization of dyes by the micelle structure also increased the filtration performance, resulting in the higher dye rejection.

Instead, cake formation took place when the saponin was added below the CMC. This was indicated by the data fitted to the cake formation fouling mechanism (*R*^2^ of 0.925). Under this condition, the solution consisted of free saponin molecules, free dye molecules, and a small amount of uncompleted surfactant micelle (pre-micelle) [36]. The free saponin molecule was an amphiphilic substance with both hydrophobic and hydrophilic parts, while the PES membrane used in this study tended to be hydrophobic. Because of this characteristic, some of the dye was attached to the saponin structure and the hydrophobic part of the saponin structure was attached to the membrane surface. On the membrane surface, the concentration of solute (saponin and dye) increased, inducing a concentration polarization [34]. Hence, when the solution was filtered by the PES membrane, the molecule was deposited on the membrane surface, causing fouling over the time of filtration, and inducing membrane pore blocking. The phenomena have a relation to the continuously decreasing flux profile along the filtration due to the sustained cake fouling discussed in point 3.1. Considering this blocking mechanism, the MEUF of dye wastewater, using saponin as the surfactant, should be conducted with the saponin concentration above its CMC.

**Figure 6:**
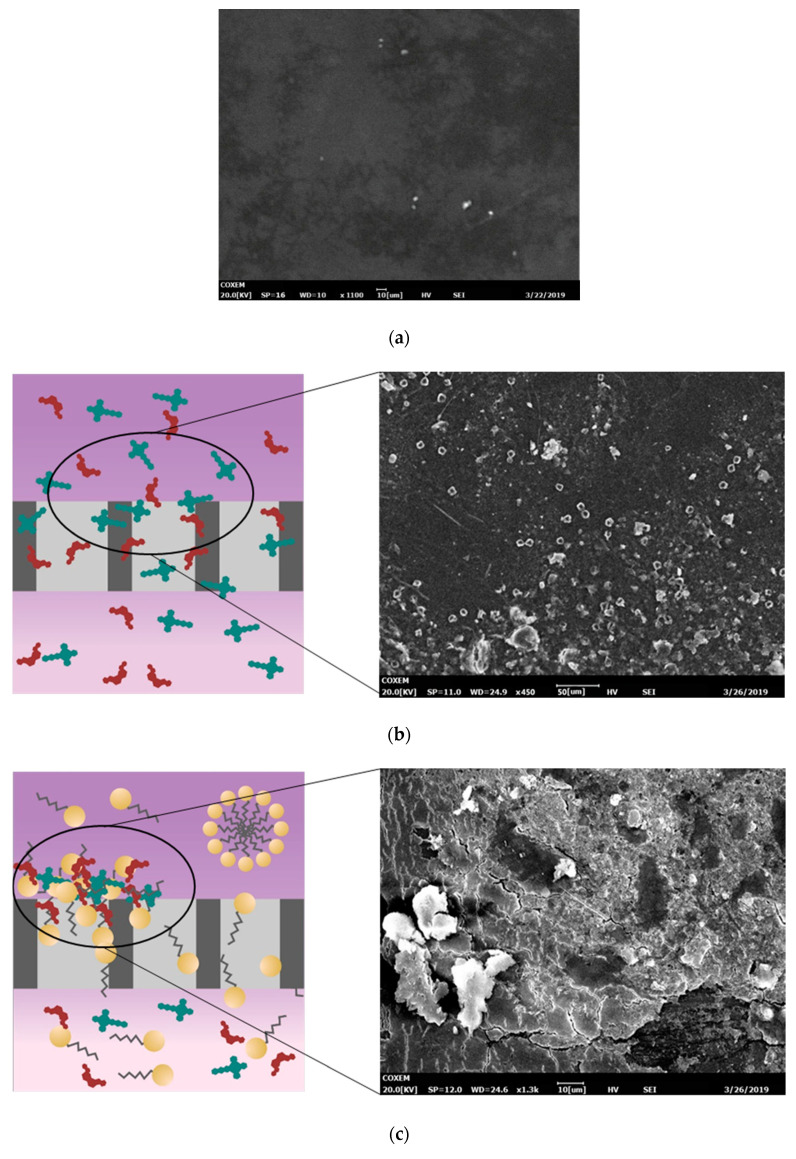

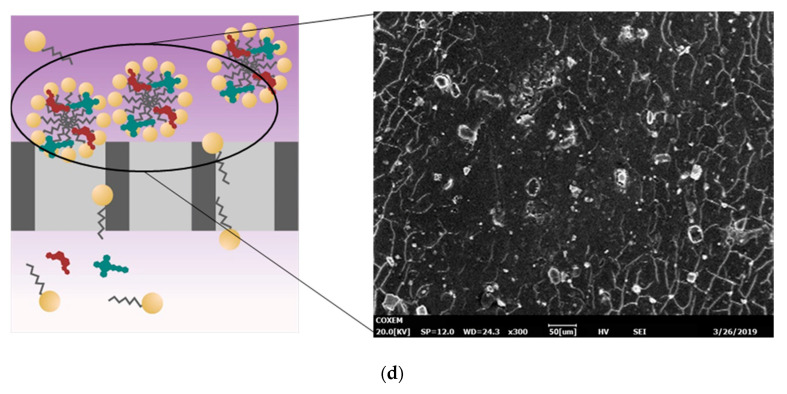
Illustration of membrane blocking and membrane surface SEM images of (**a**) clean membrane, and fouled membrane, (**b**) without saponin, (**c**) with saponin under CMC, and (**d**) with saponin above CMC.

Determination of the fouling mechanism was also confirmed by the FTIR and SEM analysis. SEMs of clean and fouled membranes as well as the illustration of membrane blocking based on the formation of surfactant micelle are shown in Figure 6. This figure shows that the used membrane was fouled compared to the clean membrane. The thick layer of cake fouling was identified from the surface of the membrane used to filter the feed with a saponin concentration under CMC. This result corresponded to the modeling calculation claiming that the process with saponin addition under CMC concentration showed cake formation fouling, while the other fouled membrane showed a thinner fouling. However, different types of fouling were found in the SEM analysis. The fouled membrane without the addition of saponin showed organic fouling, similar to that found in previous research by Hu et al. [37]. This was anticipated, as the dye wastewater model was made using Remazol dye, which is organic. On the other hand, the addition of saponin to the feed yielded colloidal fouling. Colloidal fouling refers to a fouling of the membrane surface caused by the colloids or particles deposited on the membrane materials [38]. One common colloidal fouling is formed by organic macromolecules in the feed solution, such as polysaccharides [39]. Saponin, being a polysaccharide surfactant, can deposit colloidally on the membrane surface. According to the modeling calculation, the addition of saponin above the CMC will change the fouling mechanism from cake formation to complete blocking. SEM analysis showed compatible results, wherein the membrane used to filter wastewater, with the addition of saponin above the CMC, showed less fouling.

**Figure 7:**
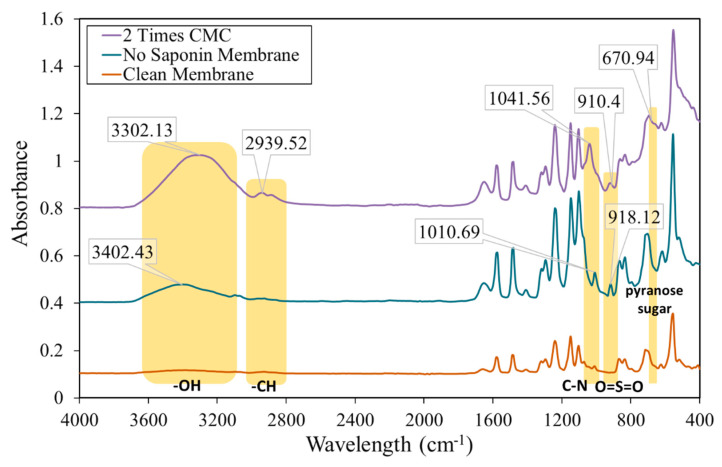
FTIR Spectra of clean polyethersulfone (PES) and fouled membranes.

FTIR analysis was also conducted to confirm the membrane fouling mechanism. Figure 7 shows the FTIR spectra of clean membrane and fouled membrane. Generally, the spectra of both fouled membranes showed similar spectra to the clean membrane. However, the fouled membrane without the addition of saponin showed peaks of C–N and O=S=O at 1010.69 cm^−1^ and 918.12 cm^−1^, respectively. The peaks reflected the specific functional group of Remazol Blue as the foulant. A weak peak at 3402.43 cm^−1^ was also assumed to be the small amount of –OH group from the Remazol Blue. 

For the fouled membrane with the addition of saponin, peaks for C–N and O=S=O also showed at 1041.56 cm^−1^ and 910.4 cm^−1^, respectively. Similar with the peaks of saponin-free fouled membrane, those peaks reflected the structure of the Remazol Blue dye. Other broad peaks of –OH, peaks of –CH stretch vibration, and a weak broad peaks of pyranose sugar appeared at 3302.13 cm^−1^, 2939.52 cm^−1^, and 670.94 cm^−1^ respectively; these reflected the functional group of saponin molecules. As expected from the modeling calculation, whether or not in the presence of saponin, the Remazol dye remained as a foulant on the membrane surface. The addition of saponin to the ultrafiltration feed also showed additional saponin foulant. As the micelle functional group was not identified, the saponin foulant was identified as the saponin monomer.

## 4. Conclusions

Two kinds of Remazol dyes were successfully removed from the water solution using an ultrafiltration membrane assisted by surfactant micelle formation. MEUF performance analysis showed that the highest flux profile was achieved without the addition of any saponin, although the rejection percentage of dye was very low. On the other hand, the addition of surfactant slightly decreased the flux value, but significantly increased the rejection percentage of dye molecules. The highest rejection percentages of dye were 97.02% and 99.42% for Remazol Red and Blue, respectively, at a saponin concentration 2 × CMC. A very low *L_m_* was found for the solution with saponin below CMC, with both of the Remazol dyes. At the CMC, micelle loading increased significantly. However, the further addition of saponin led to a decrease in *L_m_* to 0.034 mM of dyes/mM of saponin and 0.043 mM of dyes/mM of saponin for Remazol Red and Blue, respectively. The SEM and FTIR analyses prove that the membrane started to become fouled, whether in the presence of saponin or not, in the feed solution. The fouling mechanisms were as follows: for the UF process, standard blocking; for the UF process with the addition of saponin to below CMC, cake formation blocking; and, with the addition of saponin to above CMC, complete blocking.

## Figures and Tables

**Table 1:** Linearization equation of blocking/fouling models based on Hermia’s model.

Model of Blocking Mechanism	Linearized Equation	Physical Concept	Eq. Number
Complete Blocking	$\ln J=\ln J_{0}-K_{c}\cdot t$	Formation of surface deposit	(7)
Standard Blocking	$\frac{1}{\sqrt{J}}=\frac{1}{\sqrt{J_{0}}}+K_{s}\cdot t$	Pore blocking and surface deposit	(8)
Intermediate Blocking	$\frac{1}{J}=\frac{1}{J_{0}}+K_{i}\cdot t$	Pore constriction	(9)
Gel/Cake Formation	$\frac{1}{J^{2}}=\frac{1}{J_{0}^{2}}+K_{ef}\cdot t$	Pore blocking	(10)

**Table 2:** Concentration of dye and saponin on the permeate after membrane separation.

Surfactant Concentration	Dye Concentration on Permeate (ppm)		Saponin Concentration on Permeate (ppm)	
	Remazol Red RB	Remazol Blue Tq	Remazol Red RB	Remazol Blue Tq
0 times CMC	187.73	170.75	0	0
0.5 times CMC	148.58	100.03	2246.25	2160.63
1 times CMC	10.34	3.83	471.75	535.25
1.5 times CMC	9.52	2.02	573.38	619
2 times CMC	8.93	1.74	611.89	639

**Table 3:** Effect of saponin concentration on the equilibrium distribution constant and micelle loading for Remazol Red and Blue.

Saponin Concentration	Micelle Loading (*L_m_*) (mM/mM)		Equilibrium Distribution Constant (*K_d_*) (mM/mM)	
	Remazol Red RB	Remazol Blue TQ	Remazol Red RB	Remazol Blue TQ
0.5 times CMC	0.002	0.006	2.079	4.187
1 times CMC	0.068	0.055	43.263	134.556
1.5 times CMC	0.041	0.047	47.068	256.336
2 times CMC	0.034	0.043	50.249	297.960

**Table 4:** Mathematical model parameter of ultrafiltration and MEUF blocking phenomena on indigo sol dye removal.

Remazol Dye	CMC	Complete Blocking (n = 2)		Intermediate Blocking (n = 1)		Standard Blocking (n = 3/2)		Cake Formation (n = 0)	
		R^2^	Kc	R^2^	Ki	R^2^	Ks	R^2^	Kfc
Red RB	0 (UF)	0.923	−0.004	0.924	1 × 10^−4^	**0.952**	**1 × 10^−4^**	0.921	3 × 10^−7^
	0.5	0.878	−0.003	0.887	1 × 10^−4^	0.888	2 × 10^−4^	**0.912**	**1 × 10^−6^**
	1	**0.887**	**−0.004**	0.855	4 × 10^−4^	0.868	4 × 10^−4^	0.870	1 × 10^−5^
	1.5	**0.876**	**−0.004**	0.871	3 × 10^−4^	0.872	3 × 10^−4^	0.829	4 × 10^−6^
	2	**0.901**	**−0.011**	0.814	3.5 × 10^−3^	0.888	2.2 × 10^−5^	0.690	7 × 10^−4^
Blue TQ	0 (UF)	0.869	−0.002	0.870	9 × 10^−5^	**0.897**	**9 × 10^−5^**	0.863	3 × 10^−7^
	0.5	0.910	−0.006	0.918	3 × 10^−4^	0.918	3 × 10^−4^	**0.925**	**3 × 10^−6^**
	1	**0.876**	**−0.004**	0.849	4 × 10^−4^	0.857	4 × 10^−4^	0.776	2 × 10^−5^
	1.5	**0.914**	**−0.004**	0.900	3 × 10^−4^	0.912	3 × 10^−4^	0.896	6 × 10^−6^
	2	**0.975**	**−0.003**	0.948	2 × 10^−4^	0.948	2 × 10^−4^	0.942	4 × 10^−6^
